# Advances in Molecular Research on Hip Joint Impingement—A Vascular Perspective

**DOI:** 10.3390/biom14070784

**Published:** 2024-06-30

**Authors:** Riana Maria Huzum, Marius Valeriu Hînganu, Bogdan Huzum, Delia Hînganu

**Affiliations:** 1 Department of Radiology, Faculty of Medicine, “Grigore T. Popa” University of Medicine and Pharmacy, 400347 Iasi, Romania; riana-maria.huzum@umfiasi.ro; 2 Department of Morpho-Functional Sciences I, Faculty of Medicine, “Grigore T. Popa” University of Medicine and Pharmacy, 400347 Iasi, Romania; hinganu.delia@umfiasi.ro; 3 Department of Orthopedics and Traumatology, Faculty of Medicine, “Grigore T. Popa” University of Medicine and Pharmacy, 400347 Iasi, Romania; bogdan.huzum@umfiasi.ro

**Keywords:** hip joint, cellular senescence, joint labrum, *vascular endothelial growth factor (VEGF*), vasculodegenerative diseases

## Abstract

With the rise in longevity within the population, medicine continues to encounter fresh hurdles necessitating prompt actions, among which are those associated with hip joint aging. Age-related arthropathies encompass damage to bones’ articulating extremities and their supporting structures, such as articular cartilage, and alterations in the quantity and quality of synovial fluid. This study aims to summarize the biomolecular methods of hip joint evaluation focused on its vascularization, using data correlated with biomolecular research on other joints and tissues, in order to reach an objective opinion of the study prospects in this field. Following a retrospective study on most modern biomolecular research methods on the synovium, the capsule, and the articular cartilage of the hip joint, we have hereby concretized certain future research directions in this field that will improve the qualitative and morphofunctional management of the hip joint at an advanced age, even within population categories at risk of developing various degenerative joint pathologies.

## 1. Introduction

As life expectancy increases among the populace, medicine continues to face new challenges demanding swift responses, among which are the hip joint phenomena, which appear with age. Senescent arthropathies include lesions of the articular ends of the bones, their retention means, and the articular cartilage and the quantitative and qualitative changes in synovial fluid secretion [1,2,3].

Leonard Hayflick and Paul Moorhead first described the phenomenon of “cellular senescence” in 1961 [4], associating this phenomenon with apoptosis. It was once believed that this was simply an in vitro phenomenon due to the culture shock of cell phones [5]. At present, cellular senescence is considered a signal transduction process by which cells enter a stable state of growth arrest, remaining metabolically active but losing their ability to divide [6,7,8,9,10].

Senescence appears to be characterized by an association with a collection of cellular phenotypes that often coexist in a stressed cellular environment, with stressors ranging from altered morphology, chromatin structure, and gene expression patterns to an activated deoxyribonucleic acid (DNA) damage response [11,12,13]. In these cells, there are high levels of inflammatory cytokines, growth factors, and other factors generically known as the senescence-associated secretory phenotype (SASP) [14,15] or the senescence message secretome (SMS) [16]. The SASP can cause damage to the surrounding tissue [17,18], altering its microenvironment and regeneration [19,20].

Other stimuli/cell stress factors that trigger these phenomena are oncogene activators (radiation, oxidative stress, shortened telomeres, and unprogrammed DNA replication) or normal physiological processes, such as wound healing [21,22,23,24].

Recent studies have linked cellular senescence to the complex phenomena of aging [25] and age-related pathologies [26].

Current research suggests that the accumulation of senescent cells triggered by various stresses in the joints contributes to the pathogenesis of age-related diseases. Replicative senescence refers to a reduced replicative capacity caused by telomere shortening, while premature senescence, defined as cell cycle arrest, is induced by oxidative stress and DNA damage [27]. The characteristics of this type of cellular senescence include the increased expression of *p21, p16*, and *p53*, increased senescence-associated β-galactosidase (SA-β-Gal) activity, and high levels of reactive oxygen species (ROS) [28]. Thus, the halting of *p16* suppression causes early cellular senescence, while *p53, p21 (CIP1),* and *p15 (INK4b*) increase in pre-senescent cells. Raising the level of B-cell lymphoma-extra large (Bcl-XL) in pre-senescent cells enhances their anti-apoptotic capacity, which causes cells to progress towards senescence rather than apoptosis [29]. It has been found that the combination of mitogenic stimulation and DNA damage induces chondrocyte senescence and the increased production of senescence markers SA-β-Gal, *p16*, and the gamma phosphorylated form of the histone H2AX (γH2AX).

In replicative senescence, telomeres gradually shorten and lose their protective function of DNA stability, leading to aging [30].

Mammals such as mice, baboons, and humans are known to accumulate senescent cells as they grow older [31,32], which contributes to both aging and the development of age-related diseases [25,33]. In a different study, researchers observed the presence of senescent endothelial-like cells, vascular smooth-muscle cells, and macrophage-like cells in mice with induced atherosclerosis [34]. It is now known that senescent vascular endothelial cells are present in human atherosclerotic lesions and contribute to the development of atherogenesis [35,36]. In the context of hip joint pathology, senescent cells have been previously observed in the vicinity of osteoarthritic (OA) lesions but not in intact cartilage from the same patients or normal donors [37,38]. Moreover, transplanted senescent cells can induce an OA-like state in mice [39].

Even if the morphological and functional similarities in the temporomandibular joint structures in humans and the equine model create an opportunity to track disease progression and response to treatment, the majority of the biomarkers of cartilage degradation have not been studied so far, nor have any of the main signaling pathways, [40].

In the case of the SASP, its characteristic is the increased production of *vascular endothelial growth factor (VEGF),* a signal protein that promotes the formation of blood vessels through the processes of vasculogenesis. *VEGF* and its cognate receptors are expressed in OA cartilage and may contribute to osteogenesis dysfunction and osteophyte formation [41,42].

Matrix metalloproteinases (MMPs) are one of the most important families of proteases involved in the tight control of extracellular remodeling over time. The remodeling of the extracellular matrix (ECM) is one of the most important functions performed by these proteins, essential in the regulation of critical events such as embryonic development or tissue homeostasis. Thus, the dysregulation of any protease function that affects ECM homeostasis can contribute to the aging process. Chondrocyte SASPs include the production of matrix-degrading proteases, including matrix metalloproteinases *MMP-1* and *MMP-13* [43]. The latter appears to be essential for the irreversible degradation of the type II collagen network of cartilage in OA, in part based on its exogenous expression or deficiency [44].

Collagen represents one of the main families of ECM proteins, thus playing a critical role in maintaining the structure of most tissues. The essential functions of these proteins have been supported by the large number of genetic disorders caused by mutations in collagen genes [45]. The importance of collagen in longevity is further supported by the finding that reduced insulin/*IGF-1*(insulin-like growth factor 1) signaling—which extends the lifespan in various species—increases the expression of collagen and other ECM components in *C. elegans* worms [46].

Unlike collagen, mature elastin—an insoluble ECM protein that provides strength and elasticity to tissues such as arteries, lungs, ligaments, and the skin—has remarkable metabolic stability over time, unlike other ECM proteins [47,48]. It is also exposed to various changes during aging, and elastosis—the accumulation of partially degraded elastin fibers in the ECM—is a hallmark of vascular aging [49].

The disturbance of the balance between proteases and their inhibitors contributes to the reduction in the amount of elastin, which in turn increases vascular stiffness and reduces the resistance of these tissues [50].

Together, these changes may contribute to the development and progression of various pathogenic conditions that further compromise joint vasculature function.

This study aims to summarize the biomolecular methods of hip joint evaluation focused on its vascularization.

## 2. Materials and Methods

For this study, the following international databases were consulted: Web of Science, PubMed (National Library of Medicine and National Institute of Health), SciELO (Scientific Electronic Library Online), Springerlink, Science Direct, ResearchGate, Wiley Online Library, Lippincott Williams & Wilkins, MDPI, and Bond University Research Portal. Studies were selected based on a keyword search and admission/exclusion criteria. With the exception of Web of Science, 39 articles related to our topic were found within these databases.

We performed a categorical analysis, heterogeneity checks, a publication bias analysis, and subgroup analyses, picturing the various biomolecular techniques. We considered the following keywords as admission criteria for the studies: hip joint molecular research, labral molecular research, hip joint capsule research, and cellular matrix senescence. The document types considered were original and review articles belonging to the following Web of Science categories: surgery, rehabilitation, cell tissue engineering, developmental studies, evolutionary biology, anatomy morphology, and microscopy. We excluded any meso-level citation topics other than orthopedic surgery, musculoskeletal disorders, cell biology, vasculodegenerative diseases, molecular and cell biology, genetics, and imaging and tomography. We considered original articles and reviews published over a period of 5 years (2019–2024).

We further narrowed the search area on Web of Science to articles on cellular biology, molecular biology, biomolecules, tissue engineering, the extracellular matrix, cellular differentiation, cellular senescence, and anatomy. We only selected articles in English and found 64 publications—55 original articles and 9 reviews.

**Figure 1:**
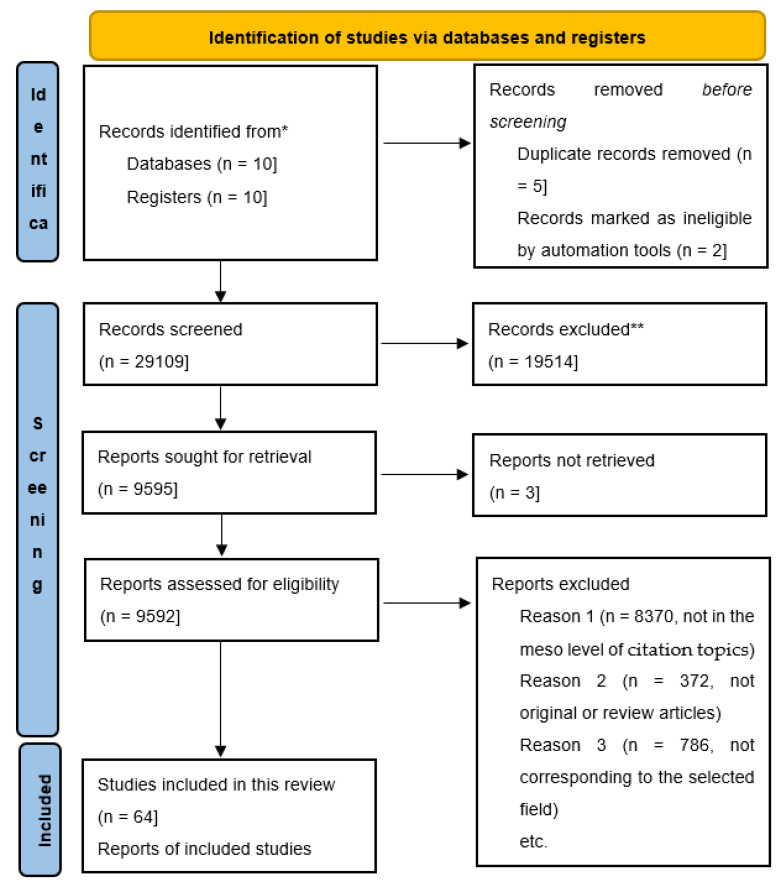
PRISMA 2020 flow diagram, including only database and register searches (https://www.prisma-statement.org/, accessed on 1 January 2020). * Records identified from databases mentioned in methods. ** Records excluded acording to selection criteria from the methods.

We found no duplicates for rejection. Article selection was performed on 15 April 2024. (Figure 1)

Finally, we conducted our research on 103 articles related to our topic.

We divided these articles into three groups:

I. Five years of molecular research on hip joint labrum, capsule, and synovial cellular senescence, with a total of 31 articles;
II. Related research on similar molecular markers, totaling 51 articles;
III. Perspectives, comprising 21 articles.

### 2.1. Five Years of Molecular Research on Hip Joint Labrum, Capsule, and Synovial Cellular Senescence

The hip joint is involved in various pathologies, starting from congenital ones and ending with changes related to senescence. The degenerative pathology of this joint includes all aspects related to the qualitative and quantitative degradation of the bone, capsule, ligament, and articular cartilage components. The starting point of these degenerative phenomena are functional or metabolic imbalances acquired or inherited by patients. Both the degradation of articular surfaces and their retention means are triggered or realized by changing the amount and constituents of the blood supply [51].

Articular components are studied separately by researchers. Thus, it was necessary to subdivide this group into three main directions of study—labral, capsular, and synovial—all finally converging towards joint ends’ bone damage.

#### 2.1.1. Hip Joint Labral Research

The acetabular labrum is a piece of fibrous cartilage to which the transverse acetabular ligament attaches anteriorly and posteriorly. The demarcation is clear, given by a distinct transition area [52]. Its triangular cross shape seals the acetabulum against the soft tissue around the femoral head joint.

This cartilage is made up of perpendicularly oriented collagen fibers up to the junction with the transverse ligament in all regions except for the anterosuperior region, where it is parallel [53]. These parallel fibers represent a weak area where most labral tears occur. Inside, this fibrocartilage is rich in nerve fibers for both nociceptive and proprioceptive functions [54]. The presence of increased nerve density in the labrum explains the pain associated with its degeneration [55].

The vascular supply of the labrum originates from the superior and inferior gluteal arteries as well as from the medial and lateral femoral circumflex arteries [55]. These vessels form a periacetabular vascular ring, with a slightly higher concentration posteriorly than anteriorly. At the same time, the vascular density has been found to be higher towards the capsular part compared to the articular part [56].

The same transition zone has been shown to be the last to heal in the case of trauma, which further reinforces the idea that it is a relatively hypovascular zone. The dimensions of the labrum vary by bone morphology function [57].

The function of the labrum is to create negative pressure in the joint by sealing the liquid in the central compartment [58], creating a suction effect which increases hip stability. Thus, it helps regulate the amount of fluid that it moves in and out of the central hip compartment and maintains a very thin layer of fluid between the femoral head and the weight-bearing acetabulum, with a much lower contact force and a lower coefficient of friction between the cartilage surfaces [59].

The term dysplastic acetabuli implies increased shear forces in the anterior and lateral bearing areas due to instability, which creates an overload on the labrum and requires hypertrophy for stability. This is the reason why hypertrophic labrums are more prevalent in the dysplastic population, supported by several studies that analyzed magnetic resonance imaging (MRI) measurements of acetabular coverage and labrum size [60].

Furthermore, labral tears are most frequently located in the anterosuperior angle of the acetabulum, an aspect usually associated with joint instability. Anterior labral tears (“at three o’clock”) can be caused by tension of the iliopsoas muscle tendon against the anterior capsulolabral structures, causing the latter to break, with a rotational movement of the hip [61].

Posterior tears are much less common and can be caused by posterior instability injuries or hip impacts. Labral tears can be classified according to etiological (traumatic, degenerative, idiopathic, or congenital) and morphological criteria (radial flap, fibrillated, longitudinal peripheral, or unstable characteristics) [62]. The Seldes classification is based on cadaveric dissection and a histological review of the labrum. It was then adapted to be used in arthroscopy, based on the morphological characteristics of the tear observed at the time of intervention: type I, when the chondrolabral junction has been disrupted; type II, when there is an intralabral tear; and type III, created by surgeons and called a combined tear as it combines the characteristics of both of the previously mentioned types [63,64].

According to recent data, molecular studies focused on the labrum targeted *VEGF*, *nerve growth factor (NGF*), and collagen [55].

A recent study showed the construction of the collagen fibrils of the acetabular labrum under light microscopy followed by scanning electron microscopy (SEM). The cartilage tissue consists of chondrocytes and type II collagen, with a layer of type I collagen. In healthy adults, chondrocytes have a rich cytoplasm and are surrounded by a dense network of fine fibrils of type II collagen and small bundles of type I collagen. In senescence, chondrocytes atrophy, and type I and II collagen fibrils are rare. Where present, the density of their chondrocytes decreases, and the cell shape and architecture of the collagen fibrils change. The cartilage has three to five layers, consisting of type I and type II collagen fibrils with a solid cartilage substrate [65].

The association between *VEGF* expression and OA is already known [66,67], while that between *VEGF* expression and acetabular labrum hyperplasia remains unclear. Labral vascular flow is related to *VEGF* expression, and the labrum has an abundance of free nerve endings and sensory nerves involved in the perception of hip pain [68,69].

Moreover, there is a correlation between the increase in *VEGF* and *NGF* values, the latter being involved in the development of nerve endings [70]. Recent studies have shown that angiogenesis contributes to the exacerbation of pain in patients with OA [71]. There is evidence of the association of increased *VEGF* and *NGF* expression with pain, reflected in studies using animal models [72,73].

In humans, *VEGF* expression in the synovial membranes of patients with painful knee OA has been demonstrated, but there are very few studies correlating the expression of *VEGF* and *NGF* in the acetabular labrum of patients with painful hip OA [74].

Finally, the molecular studies carried out over the last 5 years on the acetabular labrum investigated aspects related to the expression of type I and II collagen, *VEGF*, and *NGF*. Some of these studies are morphohistochemical, associated with imaging and/or ultrastructural aspects, but most of them address the correlation between the morphohistological and clinical aspects and therapeutic possibilities. In this research direction, the main aim is joint pain therapy (Table 1).

The data obtained from these reviews and experiments indicate that the acetabular labrum plays an important and unique role in hip joint stability, primarily supporting lateral stability during hip flexion and abduction, and is associated with robust inflammatory, degradative, immune cell recruitment, and anabolic responses during early degeneration, followed by a pronounced anti-degradative response in late degeneration. Subsequent experiments used cells from canine DDH tissues to investigate mechanobiology-related responses prior to hip osteoarthritis onset. Labral cells produce significant amounts of inflammatory and immune cell recruitment proteins in response to overload [84].

Patients presenting with degenerative phenomena of the hip joint have significantly different serum and urinary biomarker profiles compared to control groups with healthy hips, ranging from increased inflammatory biomarkers to a decreased bone metabolism, degradation, and the appearance of anabolism biomarkers. To conclude, the acetabular labrum plays a key biomechanical and biological role in maintaining joint homeostasis and responding to the pathomechanisms involved in the development and degeneration of joint pathologies. N- and C-telopeptides are still present in the mature type II collagen secreted and incorporated into cartilage [45]. MMPs, including collagenases and gelatinases, are the main “players” in type II collagen degradation [55].

The marker of type II collagen degradation (Coll2-1 biomarker) is another cartilage degradation marker that has been tested, which contains a tyrosine residue susceptible to nitration, resulting in the release of the Coll2-1-NO2 neo-epitope. Studies have shown that increased levels of oxidative and inflammatory stress in OA and rheumatoid arthritis increase the levels of these biomarkers and could provide a more specific reflection of the molecular pathways [80].

The collagen two marker (C2M) biomarker was first identified by a mass spectrometric analysis of the fragments generated by *MMP-9* cleavage from healthy human cartilage. Serum C2M is higher in patients with radiographically determined OA compared to healthy controls, but is more associated with rheumatology [82].

uCTX-II is probably the best described type II collagen biomarker and can distinguish between patients with OA and healthy controls, as well as between patients with OA with slow and rapid disease progression [75].

Aggrecan is the main ECM glycoprotein of articular cartilage and has been previously modified with chondroitin sulfate. It binds to hyaluronic acid and proteins, creating a hydrated gel that provides cartilage resistance to compression. It is composed of three globular domains (G1, G1, and G3), resembling the epidermal growth factor, complement-binding proteins, immunoglobulin folds, and proteoglycans [84].

*VEGF*-induced blood vessel proliferation occurs early on and throughout the course of degenerative hip joint diseases, allowing the *VEGF*-related gene sequence to act as a biomarker in the field of early diagnosis and disease monitoring [66].

#### 2.1.2. Hip Joint Capsule Research

The capsule of the hip joint is a strong and dense containment structure of the bone ends involved in the hip joint. It is attached 5–6 mm above the acetabular rim and the labrum, near the acetabular notch, and reinforced by the transverse acetabular ligament and the adjacent edge of the hip joint. The insertion at the level of the anatomical femoral neck is anterior to the intertrochanteric line, and posteriorly it is 1 cm above the intertrochanteric ridge.

The capsule is thicker anterosuperiorly, where maximum stress occurs, especially during standing, and posteroinferiorly it is thin and weakly attached. Its components include two types of collagen fibers—circular (orbicular zone) and longitudinal [86].

The characteristics of the ligaments attached to the capsule and their contributions to hip biomechanics and etiopathogenic processes are evolved. In the literature, how the hip capsule is managed during surgery (conservation and arthroplasty) and its effects on joint function are increasingly highlighted. The ligaments are mainly composed of collagen type I (85%) and combinations of types III, V, VI, XI, and XIV (15%). In the hip joint, higher proportions of collagen type III in the ligament capsule are associated with hip instability, while high levels in the labrum are associated with progressive joint degeneration [87,88]. The dimensions of capsular ligament tissues (i.e., thickness and length) can change, thus adapting to various pathologies [89,90,91] (Table 2).

Recent works in the literature have speculated that capsule factors may play a critical role in clinical outcomes. Lower collagen I expression has been observed in patients with a higher degree of congenital hip dislocation, suggesting that collagen I is related to the degree of dislocation and that a lower expression of collagen I may lead to joint laxity and, subsequently, a higher degree of dislocation [93].

Most of the studies regarding the hip joint capsule are observational and performed by means of dissection or MRI/CT techniques [94,95,96,97].

In other recent studies, *osteoblast-specific transcription factor (Cbfa1*) and *osteocalcin* (*OC*) genes have been shown to be expressed in osteoblasts but not fibroblasts. *Cbfa1* has a major role in the regulation of osteogenic differentiation since it is a critical transcription agent and has a role in the regulation of additional differentiation markers by promoting bone differentiation and production, meaning that it could be a marker of fibroblast osteogenic differentiation. *OC* plays the role of a non-collagenous matrix protein present in bone and is associated with bone minerals [92].

Osteogenic differentiation can be identified based on osteoblast-specific secretory proteins and the ability to mineralize the extracellular matrix. Alkaline phosphatase (ALP) is a matrix protein integrated by osteoblasts and reflects type I collagen, with the ability to transform into a mineralized stage [92,98].

#### 2.1.3. Hip Joint Synovium Research

The synovial membrane is the innermost layer of the joint capsule. In the case of the hip joint, it consists of three layers. The intimal lining layer is found closest to the joint cavity and consists mainly of macrophages (“type A cells”) and fibroblasts (“type B cells”) that show low degrees of cell division. Then follows the vascularized subintimal layer, also referred to as the sublining interstitial tissue, and, finally, a fibrous stromal layer forming the joint capsule [99]. The quantitative and, especially, qualitative microanatomy of the human articular synovium is important for understanding its physiological role but also the clinical performance of the joint replacement devices used to restore degenerated and painful joints’ function. The lubrication of bony, cartilaginous, ligamentous, and fibrous structures is provided by a fluid produced by a specialized layer of cells found on the surface of a delicate tissue, called the synovial lining, which is organized in two layers: the intima, which overlaps a vascular subintimal layer, and the fibrous stroma, i.e., the joint capsule [100,101].

This fluid controls the joint environment, participating in immunological reactions to microbes, phagocytosis (removal of detritus material), lubrication, and cartilage nutrition [102]. Its intimal layer is thick and composed of type A cells, similar to macrophages in form and function, and secretory type B cells, similar to fibroblasts [103]. The fact that this layer shows low degrees of cell division suggests that many cells in the lining migrate to the synovial surface from underlying blood vessels and are derived from bone marrow [104].

Its changes include the thickening of the intimal layer, increased vascularity, inflammatory cell infiltration of the subsynovial layer, and the formation of more villi in the joint.

Because the regenerative capacity of the synovium was unknown for a long time, the surgical removal of chronically inflamed synovial tissue from arthritic, tuberculoid, or traumatized joints was once a common orthopedic procedure. However, this regenerative property allows for the lubrication of implanted artificial joints despite surgical removal or damage to synovial tissues [105].

Metabolomic analyses of synovial fluid have identified potential biomarkers for the early diagnosis of degenerative knee pathologies and prognostic biomarkers of disease progression. Future studies will connect these biomarkers to cellular pathophysiology and help discover the metabolic process or metabolite itself causing disease progression. Currently, we know that the metabolic homeostasis of affected chondrocytes and metabolic-immune dysregulation are probable factors linking the etiopathogenesis of degenerative hip diseases to obesity and senescence.

The evidence involving cellular senescence in joint tissues as an etiopathogenic element in hip joint degenerative disease onset and progression is compelling. However, further research is needed to identify the precise mechanisms by which senescence causes specific disease phenotypes (Table 3). Most likely, the etiopathogenic connection between aging, senescence, and a degenerative pathology is the accumulation of senescent cells in the synovium and articular cartilage, combined with gradual changes in metabolism, morphology, cell function, and blood supply [106].

The common biomarkers presented to identify senescence are insufficient to diagnose a degenerative pathology. SA-β-Gal may be influenced by changes in autophagy and lysosome function. *p16* expression is not necessary for SASP pathogenesis and degenerative joint pathology. Extracellular vesicles (Evs) as well as urokinase plasminogen activator surface receptor (uPAR) expression (which is present in senescent chondrocytes) should be further investigated to determine whether they are accurate clinical markers of a degenerative joint pathology [1,106].

Synovial fluid undergoes distinct changes in response to trauma. Thus, amino acids and their metabolites are associated with end-stage osteoarthritis, but they seem to be present in increased amounts in acute lesions as well [114].

### 2.2. Related Research on Similar Molecular Markers

The vast majority of studies related to our research topic investigate clinical and tissue-engineering aspects. The role of senescent skeletal cells (chondrocytes, osteoblasts, osteoclasts, osteocytes, and muscle cells) in initiating the development and progression of degenerative hip diseases, with or without interaction with macrophages/synovial cells, should be considered. The current trend is to find a curative treatment for degenerative hip joint diseases by eliminating senescent skeletal cells and/or inhibiting the SASP that leads to senescence [42,113,116,117,118,119,120].

Recent studies have shown that toll-like receptor 4 (TLR4, a receptor located on the surface of osteoclasts and osteoblasts) plays an essential role in the development of osteoporosis, with clinical data confirming that TLR4 polymorphisms and aberrant expression are associated with osteoporosis. The disruption of osteoblast and osteoclast activity induced by abnormal TLR4 expression is the main molecular mechanism underlying the pathological processes of osteoporosis and may be associated with the interactions between the TLR4 pathway and transcription factor NF-κB, pro-inflammatory effects, non-coding ribonucleic acid (ncRNA), and runt-related transcription factor 2 (RUNX2] [36,71,121].

The SASP, together with a mild inflammatory response characteristic of senescent cells, creates the ideal preamble for the development of cardiovascular diseases such as atherosclerosis, coronary heart disease, and myocardial infarction [122,123]. It is possible that these cardiac and, especially, vascular pathologies directly affect the blood supply of the hip joint, triggering its degenerative pathology. Regarding these, glycoprotein vascular cell adhesion molecule 1 (VCAM-1) presents remarkable potential for objectifying the forms of articular cartilage and synovial fluid degeneration [81].

Most of the studies that are included in Table 1, related to the articular labrum, focus on researching the expression of specific markers of collagen, *VEGF, NGF,* and MMs. The same markers of collagen and MMs are also considered by some studies in synovial fluid and the joint capsule.

Most people affected by degenerative hip joint arthropathies have associated joint component lesions at the time of diagnosis, with the highest prevalence being at an age over 55 years among women (over 58% of the cases studied in the investigated articles).

However, there is no direct correlation between their results and their working methods are different, specific to the tissue type. The studies investigating degenerative bone damage were not taken into consideration, because it is a stage of disease that can only be treated interventionally. The results of our study highlight a direction of biomolecular research combining the outcomes of studies on the various articular components, with the aim of finding a new research direction that promises the early diagnosis, prevention, and even treatment of these pathologies.

### 2.3. Perspectives

After having analyzed the variability in the factors used for diagnosis, genetic evaluation, prevention, and treatment, we believe that there is an important connection between all of them—the microvascularization of the hip joint and its quantitative (synovium filtration microperfusion, the presence of vascular areas exposed to trauma, and the possible presence of hypovascular areas) and qualitative aspects (minimum functional flow, adaptability to traumatic stress and physical demands, and changes related to aging).

There are studies that indirectly support this theory. As in the case of other anatomical structures (e.g., the superficial musculoaponeurotic system of the face), a series of therapeutic, interventional, or medical acts can indirectly demonstrate the role of the vascular system in the etiopathogenesis of degenerative hip joint diseases.

Genicular artery embolization (GAE) represents a new method of symptomatic treatment in osteoarthritis. Inflammatory synovial disease (synovitis) can affect other joint components by altering chondrocyte function, while increased angiogenesis and bone remodeling contribute to chronic inflammation. Angiogenesis can also appear pathologically, in which case it leads to chronic inflammatory conditions. On the other hand, vasculogenesis, a consequence of angiogenesis, occurs when circulating angioblasts differentiate into endothelial cells. These new blood vessels require newly created spaces at the bone level. This is where MMPs and other cytokines intervene to establish a perivascular environment for the formation of new arterioles and venules. Within this process of “angiogenic sprouting” the increase in *VEGF* and *PDGF* expression is justified. Neo-vessels may contribute to inflammation persistence by maintaining oxygen and nutrients in abnormal endothelial cells, as well as providing pro-inflammatory cytokines access to the local microenvironment [124,125,126].

For the same purpose as symptomatic treatment, intra-articular injections with selective inhibitors of vascular endothelial growth factor receptor-1 (VEGFR1)/vascular endothelial growth factor receptor-2 (VEGFR2) kinase (pazopanib) or VEGFR2 kinase (vandetanib) have been performed; the former has been shown to immediately alleviate OA pain by interfering with the pain transmission pathways, while the latter has a somewhat similar effect, mainly due to the inhibition of cartilage degeneration by suppressing VEGFR2 expression. It has also been shown that pazopanib AI simultaneously inhibits cartilage degeneration [67].

At the same time, testing the presence of hormonal receptors at the level of the joint components will also be useful and feasible in future studies.

## Figures and Tables

**Table 1:** Articles related to biomolecular research on the acetabular labrum, in alphabetical order.

Article	Histology/Other	Biomolecule	Ultrastructural Association	IRM/CT Association/Other	H/AM	Statistics/ Scoring/ Scale	Aim
Antoniadis et al. [75]	ELISA/Explant culture/Alcian blue/Alizarin Red S	Pro/Pro-Col-Iα, IL-6, COMP/VEGF, ACAN	_	Fluorescence imaging	H	Hierarchical clustering in ClustVis 1.0/GraphPad Prism/one-way ANOVA with Tukey’s multiple-comparison test/Mann–Whitney test and Friedman’s test with Dunn’s multiple-comparison test	Putative biomarkers of OA from explanted human labrum tissues under basal and inflammatory conditions
Bay-Jensen et al. [76]	_	CTX-II/TIMP	_	_	H	_	Review of biomarkers derived from type II collagen and aggrecan
Hasan et al. [77]	_	TNFα/ TNF mRNA/TNFbeta/IL-1/IL-1b/ N-terminal-telopeptide (NTX)	_	_	H	ICROMS tool (integrated quality criteria for review of multiple study designs)	Review of blood and urine degenerative biomarkers
Huang et al. [66]	Nanoparticles	VEGF/ SsDNA/CRISPR/Cas12a	_	Fluorescence spectroscopy	H	_	Rapid detection of ssDNA molecules
Koyama et al. [78]	Real-time polymerase chain reaction (PCR)	RNA/TNFA/IL1B/IL6/COX2 mRNA	_	_	H	SPSS version 19.0	Compare synovial tissue’s inflammatory cytokine levels
Lynch et al. [79]	_	COMP	_	_	H	Fixed-effect inverse-variance model	Review of COMP
Mobasheri et al. [80]	_	Collagen	_	_	H	_	Review of glycosylation of collagens
Nogami et al. [65]	Hematoxylin and eosin (HE) and Alcian blue (AB)	Collagen I and II	Collagen I and II	_	H	_	Ultrastructure of collagen fibers
Oppl et al. [81]	ELISA—enzyme-linked immunosorbent assay	VCAM-1	_	_	H		Clinical future prediction model development
Osashi et al. [82]	PCR	RNA	_	_	H	G∗POWER3	NGF-expressing cells/mRNA expression of NGF, CD14, and CD90
Sato et al. [55]	Hematoxylin and eosin	Anti-VEGF/ Anti-NGF/ ISOGEN reagent	_	_	H	Krenn score/ Kellgren–Lawrence grade/ Harris Hip Score/ Spearman’s correlation analysis	VEGF and NGF expression levels
Schon et al. [83]	HE/ Picrosirius Red	MMPs/TIMPs	_	_	H	Mann–Whitney U test	Expression profile of matrix metalloproteinases
Stattin et al. [84]	Cell culture/Recombinant protein expression	*ACAN* gene (NM_00135.2, NM_013227.2)	_	Chemiluminescent detection and documentation (Bio-Rad ChemiDoc MP)	H	Affinity Design 1.7 software	*ACAN* variants linked to hereditary skeletal disorders
Wang et al. [85]	_	Serum 25-hydroxyvitamin D	_	_	H	Categorical analysis, heterogeneity checks, publication bias analysis, and subgroup analyses	Review 25-(H)D

Pro, proteoglycans; H, human; AM, animal model; CTX-II, urine type II collagen C-telopeptide; TIMP, tissue inhibitor of metalloproteinase-1; COMP, cartilage oligomeric protein; VCAM-1, vascular cell adhesion molecule 1.

**Table 2:** Articles related to biomolecular research on the hip joint capsule, in alphabetical order.

Article	Histology/Other	Biomolecule	Ultrastructural Association	IRM/CT Association/Other	H/AM	Statistics/ Scoring/ Scale	Aim
Jiang et al. [92]	Cell cultures/chromatography via an ALP detection kit/chromatography using a hydroxyproline detection kit/PCR	*Cbfa1α sense/Cbfa1α anti-sense/OCα sense*/*OCα anti-sense/BMPR-I/BMPR-II/Smad1/Smad5*/*phosphorylated (p) Smad1/pSmad5/Smad4/Smad6/Cbfa1*					Osteogenic disparity characteristics of fibroblasts in hip joint capsule
Li et al. [72]	Cell cycle, viability, apoptosis, immunofluorescence, reverse transcription polymerase chain reaction/RT-PCR/Western blotting	*COL1A1/COL3A1/MMP1/MMP3/MMP9/MMP13/TGF-β1/TGF-β2/SMAD3/WNT11/αSMA/CCNB1/CCNE2/CCNA2/CDK1/E2F1/CDC6/CDC7*	_	_	H	Unpaired two-tailed *t*-test with GraphPad Prism software, USA	Molecular changes in hip dysplasia
Zhang et al. [93]		Monoclonal rabbit anti-human collagen I/rabbit anti-human collagen III antibody/qPCR RT		X-ray imaging		Tonnis’ classification of hip dislocation/statistical software SPSS 16.0/two-tailed Student’s *t*-tests	Roles of collagen I and III in the hip capsule
Zhao et al. [87]	HE/MASSON	DNBelab C Series Single-Cell Library Prep Kit/Metabolic genes	_	_	H	clusterProfiler R package/pseudotemporal analysis	Receptor-like cells and ligament stem cells

H, human; AM, animal model.

**Table 3:** Articles related to biomolecular research on hip joint synovial fluid, in alphabetical order.

Article	Histology/Other	Biomolecule	UA	IRM/CT Association/Other	H/AM	Statistics/ Scoring/ Scale	Aim
Batushansky et al. [107]	Liquid chromatography/gas chromatography–mass spectrometry	Glycine/histidine, lysophospholipid LPC/hypoxanthine/homocysteine/urate/tryptophan/fructose/citrate/malate/methionine/3-hydroxybutyrate	_	_	H	BIPED classification model of OA biomarkers	Review to relate metabolic syndrome to synovium pathology
Carlson et al. [108]	High-performance liquid chromatography–mass spectrometry (LC-MS)	Glycine/serine/alanine/threonine/lysine, arginine/proline/urea cycle/phosphatidylinositol phosphate metabolism/carnitine shuttle/vitamin metabolism (B5 and C)/porphyrin metabolism	_	_	H	MetaboAnalyst/Kolmogorov–Smirnov test (KS-test)/HCA/Mummichog/Volcano plot analysis	Metabolomic phenotypes from human synovial fluid
Coryell et al. [106]	_	SA-β-Gal production/*p16* expression/EV secretion	_	_	H	_	Review of phenotypes associated with cellular senescence
Haubruck et al. [99]	_	Monocyte/Macrophage	_	_	H	_	Review of synovial in situ dynamics of joint macrophages and monocytes
Liu et al. [1]	_	GATA/STING*FOXD1/SIRT6/DGCR8*	_	_	H	_	Systematic review/chondrocyte senescence/senescent fibroblast-like synoviocytes
Kim et al. [109]	Gas chromatography/time-of-flight mass spectrometry	Hypoxanthine/xanthine/adenosine/citrulline/histidine/tryptophan	_	_	H	MetaboAnalyst	*RA and OA metabolomic differentiation*
Koyama et al. [78]	Real-time polymerase chain reaction (PCR)	*RNA*/TNFA/IL1B/IL6/COX2 mRNA	_	_	H	SPSS version 19.0	Compare synovial tissue inflammatory cytokine levels
Laus et al. [110]	1H-NMR	Tryptophan/phenylalanine/tyrosine/glycine/asparagine/glutamine/arginine/methionine/1,3-Dihydroxyacetone	_	_	NH	Box–Cox transformation/*t*-test	Metabolomic phenotypes from horse synovial fluid
MurilloSaich et al. [111]	HE/ELISA	*CD68*/*Vimentin*/TNF/*CXCL2/* *CCL2/MMP13*	_	A 600 MHz Bruker Avance III spectrometer 1 H NMR	H	Krenn OPLS-DA/VIP	Potential metabolomic biomarkers of joint injury in synovial fluid and serum
Stabile et al. [112]	1H-NMR	Isoleucine/leucine/valine/β-hydroxybutyrate/threonine/alanine/glutamine/methionine/lactate/acetate/acetoacetate/pyruvate/citrate/creatine/creatinine/β-glucose/TMAO/lactate/histidine/phenylalanine/tyrosine/formic acid	_	_	NH	R Statistic software/unsupervised PCA, HCA/supervised OPLS-DA	Metabolomic phenotypes from canine synovial fluid
Yang et al. [113]	_	RNA/RT-qPCR/synovial mesenchymal stem cells/*VEGF/MMP9*	_	_	H	R software/GraphPad Prism/Shapiro–Wilk test	Characteristics of the synovium in OA
Wallace et al. [114]	High-performance liquid chromatography	GAG monomers/steroid hormones/NADPH	_	_	NH	MetaboAnalyst/hierarchical cluster analysis	Potential metabolomic biomarkers of joint injury in synovial fluid and serum
Zhan et al. [115]	Mass spectrometry	Geniposide-related biomarkers	_	_	H	MetaboAnalyst/hierarchical cluster analysis	Potential metabolomic biomarkers of joint injury in synovial fluid

H, human; AM, animal model; RA, Rheumatoid arthritis; OPLS-DA, histopathological synovitis score/orthogonal partial least square discrimination analysis; VIP, variables important for projection; 1H-NMR, one-dimensional protonuclear magnetic resonance; UA, ultrastructural association.
